# Development and internal validation of a multivariable risk stratification model for preoperative anxiety in surgical patients: a retrospective observational study

**DOI:** 10.3389/fmed.2026.1798841

**Published:** 2026-04-21

**Authors:** Cheng Wu, Wei Li, Hanwen Fan, Min Zhang

**Affiliations:** Department of Anesthesiology, Hejiang People’s Hospital, Luzhou, Sichuan, China

**Keywords:** nomogram, preoperative anxiety, retrospective study, risk stratification, surgical patients

## Abstract

**Background:**

Preoperative anxiety is common among surgical patients and is associated with adverse perioperative outcomes. Early identification of patients at elevated risk of clinically significant preoperative anxiety may facilitate timely psychological screening, support, and targeted interventions. This study aimed to identify factors associated with preoperative anxiety and to develop and internally validate a multivariable risk stratification model for individualized preoperative anxiety assessment.

**Methods:**

This retrospective observational study included all consecutively eligible adult patients who underwent elective surgery at a tertiary teaching hospital in Southwest China between January 2021 and October 2024 and had a routinely documented preoperative psychological assessment in the medical record. Preoperative anxiety was retrospectively determined based on standardized cut-off values of validated anxiety assessment instruments documented in preoperative records. Demographic, clinical, perioperative, psychological, behavioral, and social variables were extracted from electronic medical records and routine preoperative assessments. Univariate and multivariable logistic regression analyses were performed to identify independent predictors of preoperative anxiety. Model discrimination was evaluated using receiver operating characteristic (ROC) curve analysis, calibration was assessed using calibration plots with bias correction, and a nomogram was constructed based on the final multivariable model.

**Results:**

Among 425 eligible patients, 168 (39.5%) met the criteria for preoperative anxiety. Multivariable analysis identified female sex, lower body mass index, higher ASA physical status (III–IV), longer expected operative time, poorer sleep quality, higher depressive symptom scores, night-time smartphone use for at least 1 h, longer daily smartphone use duration, and higher trait anxiety scores as independent risk factors for preoperative anxiety, whereas greater social support was independently protective. The prediction model demonstrated good discriminative performance, with an area under the ROC curve of 0.848 (95% confidence interval, 0.811–0.884). At the optimal cut-off value, the model achieved a sensitivity of 0.73, a specificity of 0.82, and an overall accuracy of 0.76. Calibration analysis showed good agreement between predicted and observed risks. A nomogram was developed to facilitate individualized risk prediction.

**Conclusion:**

This multivariable risk stratification model showed good discrimination and calibration for identifying surgical patients at risk of preoperative anxiety. The nomogram provides a practical tool for individualized preoperative risk stratification and may support early psychological screening and targeted perioperative support within similar elective surgical settings. External validation in independent multicenter cohorts is warranted before broader implementation.

## Introduction

Preoperative anxiety is a common psychological response among patients awaiting surgery and has been reported to affect approximately 20–50% of surgical populations, depending on patient characteristics, surgical type, and assessment methods (1–3). Elevated levels of preoperative anxiety have been associated with adverse perioperative outcomes, including increased anesthetic requirements, heightened postoperative pain, delayed recovery, prolonged hospital stay, and reduced patient satisfaction (4–6). Consequently, early identification of patients at high risk of preoperative anxiety has become an important component of perioperative care.

Previous studies have identified a wide range of factors associated with preoperative anxiety, encompassing demographic characteristics, clinical status, and psychosocial conditions. Female sex, younger age, lower educational level, higher surgical risk, and longer expected operative time have been reported as important correlates of preoperative anxiety, although the mechanisms underlying some of these associations—particularly those related to educational attainment—may be complex and may partly involve differences in health literacy and information processing (7, 8). Psychological and behavioral factors, such as poor sleep quality, depressive symptoms, and higher trait anxiety, are also strongly linked to anxiety before surgery (9). In addition, social support has been recognized as a protective factor, with lower perceived support associated with increased emotional distress in surgical settings (10).

In recent years, increasing attention has been paid to the role of digital behaviors in mental health. Excessive smartphone use, particularly during night-time hours, has been associated with sleep disturbance, emotional dysregulation, and anxiety symptoms in both general and clinical populations (11, 12). However, evidence regarding the relationship between smartphone use patterns and preoperative anxiety remains limited. Most existing studies have focused on single behavioral or psychological factors, rather than integrating digital behaviors with traditional clinical and psychosocial predictors in surgical patients.

Although numerous factors related to preoperative anxiety have been described, most prior research has relied on univariate analyses or focused on individual risk factors (13, 14). Few studies have developed comprehensive multivariable prediction models that simultaneously incorporate demographic, clinical, psychological, behavioral, and social variables to estimate individualized risk. Moreover, validated and clinically applicable tools, such as nomograms, for predicting preoperative anxiety in routine surgical practice are still scarce (15). This gap limits the ability of clinicians to efficiently identify high-risk patients and implement targeted interventions before surgery.

Therefore, the present study aimed to identify independent predictors of preoperative anxiety in a cohort of surgical patients and to develop and internally validate a multivariable prediction model using routinely available preoperative data. By integrating clinical characteristics, psychological symptoms, sleep-related factors, smartphone use behaviors, and social support, we sought to construct a practical nomogram to facilitate individualized risk assessment and support early psychological screening in perioperative care.

## Methods

### Study design and setting

Patients who underwent elective surgery between January 2021 and October 2024 were consecutively included, reflecting routine preoperative practice within this single tertiary center. Clinical, perioperative, and psychosocial information was retrospectively collected from electronic medical records and standardized preoperative assessment records.

### Participants

In this retrospective study, adult patients (≥18 years) who underwent elective surgical procedures during the study period were eligible for inclusion if standardized preoperative psychological assessment data were available in the medical records. Patients were excluded if they had documented cognitive impairment, severe psychiatric disorders requiring ongoing treatment, emergency surgery, or incomplete key clinical or questionnaire data. During the study period, preoperative psychological questionnaires were collected in routine clinical practice and documented in the electronic medical record as part of the preoperative assessment process when available.

All eligible patients—defined *a priori* as adult elective surgical patients with available, routinely documented preoperative psychological assessment data—were retrospectively identified from institutional records and consecutively included without any additional sampling to minimize selection bias. Preoperative anxiety status was determined retrospectively based on established cut-off values of validated anxiety assessment instruments, and patients were categorized into anxiety and non-anxiety groups accordingly.

### Variables and outcome definition

The target condition for preoperative risk stratification in this study was clinically significant preoperative anxiety, which was retrospectively determined based on standardized cut-off values of validated anxiety assessment instruments documented in preoperative evaluation records. For the purpose of clinical screening and risk stratification, anxiety status was dichotomized into anxiety versus non-anxiety groups according to these predefined, clinically validated cut-off values. Candidate predictors were prespecified according to clinical relevance and existing literature and were retrospectively extracted from electronic medical records and standardized assessment forms. Demographic variables included age, sex, body mass index (BMI), and educational level. Clinical characteristics comprised chronic comorbidities (hypertension, diabetes mellitus, and cardiovascular disease), history of previous surgery, smoking status, alcohol use, and American Society of Anesthesiologists (ASA) physical status classification. Perioperative variables included planned surgical category and expected operative time as recorded in preoperative anesthesia or surgical planning documents. Psychological and behavioral variables were obtained from routinely collected preoperative assessments and included sleep quality score, presence of insomnia symptoms, depressive symptoms measured using the Self-Rating Depression Scale (SDS), baseline trait anxiety score, and pain intensity at admission assessed using the Numerical Rating Scale (NRS). Smartphone use behaviors were assessed using standardized self-reported items routinely documented in the structured preoperative assessment records, including whether the patient reported night-time smartphone use for at least 1 h and the average daily duration of smartphone use. Social support was evaluated using a standardized social support score recorded during preoperative assessment. Health literacy was not routinely assessed using a validated instrument during the study period and therefore was not available for inclusion in the present analysis.

### Data sources and measurement

Demographic, clinical, and perioperative variables were retrospectively extracted from the hospital’s electronic medical record system. Psychological, behavioral, and social variables were obtained from standardized self-reported questionnaires that had been routinely completed by patients as part of preoperative clinical assessment and documented in the medical records. For smartphone-related variables, the routine preoperative assessment included structured items on night-time smartphone use and average daily smartphone use duration. “Night-time smartphone use for at least one hour” was coded according to the recorded response to this standardized assessment item, and daily smartphone use duration was abstracted from the same structured preoperative record. Pain intensity at admission was recorded using the Numerical Rating Scale (NRS) as part of routine clinical evaluation. All data included in the analysis were collected prior to surgery and were derived from existing records using standardized assessment instruments and uniform clinical procedures, ensuring consistency and comparability across study groups.

### Bias and study size considerations

As this was a retrospective study, eligible patients were retrospectively identified from institutional records and consecutively included to minimize selection bias. Information bias was reduced by relying on routinely collected clinical data and standardized assessment instruments documented in the medical records. Potential confounding was addressed through multivariable logistic regression analyses, in which clinically relevant variables and those identified in univariate analyses were simultaneously adjusted. In particular, variables reflecting baseline physiologic status and anticipated operative burden (e.g., ASA physical status and expected operative time), as well as major comorbidities and planned surgical category, were included as clinical covariates to partially account for differences in underlying illness severity. The study sample size was determined by the total number of consecutively eligible elective surgical patients with documented routine preoperative psychological assessments available during the study period. The number of patients meeting the criteria for preoperative anxiety provided an adequate number of target-condition events to support multivariable logistic regression modeling according to commonly accepted methodological criteria.

Because patients without documented routine preoperative psychological assessments were not included, some degree of selection bias related to data availability cannot be fully excluded.

### Statistical analysis

Continuous variables were summarized as means with standard deviations or medians with interquartile ranges, depending on data distribution, and were compared using independent-samples t tests or Mann–Whitney U tests, as appropriate. Categorical variables were expressed as counts and percentages and compared using the χ^2^ test.

Univariate logistic regression analyses were conducted to evaluate the association between each candidate predictor and dichotomized preoperative anxiety status. Variables that were statistically significant in univariate analyses or prespecified based on clinical relevance and prior literature were entered into a multivariable logistic regression model to identify independent predictors of preoperative anxiety. Only variables that retained independent predictive value in the final multivariable model were included in the final risk stratification model and the derived nomogram. Adjusted odds ratios (aORs) with 95% confidence intervals (CIs) were reported. Model discrimination was assessed using receiver operating characteristic (ROC) curve analysis, and the area under the curve (AUC) was calculated. The optimal probability threshold was determined using the Youden index. Model calibration was evaluated using calibration plots with bias correction. Based on the final multivariable model, a nomogram was constructed to facilitate individualized risk prediction. Prespecified subgroup analyses were further performed to evaluate model robustness across surgical category, ASA physical status, and sex, with discriminative performance assessed using subgroup-specific AUCs. All statistical analyses were performed using standard statistical software. A two-sided *p* value <0.05 was considered statistically significant.

## Results

### Baseline characteristics of the study population

The baseline characteristics of the 425 surgical patients stratified by preoperative anxiety status are summarized in Table 1. Overall, 168 patients (39.5%) met the criteria for preoperative anxiety, while 257 patients (60.5%) did not. Patients with preoperative anxiety were significantly younger than those without anxiety (49.1 ± 12.8 vs. 54.6 ± 13.1 years, *p* < 0.001) and were more likely to be female (66.7% vs. 49.0%, *p* = 0.001). The anxiety group also had a lower mean body mass index (BMI) compared with the non-anxiety group (22.9 ± 3.4 vs. 24.2 ± 3.7 kg/m^2^, *p* = 0.002). Educational attainment differed significantly between groups (*p* = 0.008), with a lower proportion of patients with college-level education or above among those with anxiety. Regarding comorbid conditions, patients with anxiety had lower prevalences of hypertension (25.0% vs. 37.7%, *p* = 0.006) and cardiovascular disease (8.9% vs. 15.2%, *p* = 0.048), but were more likely to report a history of previous surgery (46.4% vs. 36.2%, p = 0.04). No significant differences were observed in diabetes mellitus, smoking status, or regular alcohol use between the two groups. From a perioperative perspective, patients with preoperative anxiety were more likely to have a higher ASA physical status (III–IV) than those without anxiety (41.7% vs. 24.1%, *p* = 0.015). This difference suggests that clinical risk profiles and anticipated operative burden differed between groups, underscoring the need for multivariable adjustment when assessing associations with preoperative anxiety. Although the distribution of planned surgical categories did not differ significantly, the anxiety group had a longer expected operative time (median 180 vs. 150 min, *p* = 0.003). Psychological, behavioral, and social factors showed marked differences between groups. Patients with anxiety reported poorer sleep quality (8.1 ± 3.0 vs. 5.9 ± 2.8, *p* < 0.001) and a higher prevalence of insomnia symptoms (44.6% vs. 28.0%, p < 0.001). They also had higher depressive symptom scores, higher pain intensity at admission, more frequent night-time smartphone use (≥1 h), and longer daily smartphone use (all *p* < 0.01). In contrast, social support scores were significantly lower in the anxiety group (31.2 ± 6.9 vs. 35.5 ± 7.0, *p* < 0.001). As expected, baseline trait anxiety scores were substantially higher among patients with preoperative anxiety than among those without anxiety (48.9 ± 9.2 vs. 37.6 ± 7.8, p < 0.001).

**Table 1:** Baseline characteristics of the study population stratified by preoperative anxiety status (*N* = 425).

Characteristics	Total (*N* = 425)	Anxiety (*n* = 168)	No anxiety (*n* = 257)	*p-*value
Age, years (mean ± SD)	52.4 ± 13.2	49.1 ± 12.8	54.6 ± 13.1	<0.001
Female, n (%)	238 (56.0)	112 (66.7)	126 (49.0)	0.001
BMI, kg/m^2^ (mean ± SD)	23.7 ± 3.6	22.9 ± 3.4	24.2 ± 3.7	0.002
Education level, *n* (%)	0.008			
Primary or below	118 (27.8)	59 (35.1)	59 (23.0)	
Secondary school	206 (48.5)	77 (45.8)	129 (50.2)	
College or above	101 (23.8)	32 (19.0)	69 (26.8)	
Chronic comorbidities, *n* (%)				
Hypertension	139 (32.7)	42 (25.0)	97 (37.7)	0.006
Diabetes mellitus	68 (16.0)	21 (12.5)	47 (18.3)	0.11
Cardiovascular disease	54 (12.7)	15 (8.9)	39 (15.2)	0.048
Previous surgery	171 (40.2)	78 (46.4)	93 (36.2)	0.04
Smoking status, *n* (%)	0.27			
Current smoker	59 (13.9)	19 (11.3)	40 (15.6)	
Alcohol use, *n* (%)	0.31			
Regular drinking	48 (11.3)	14 (8.3)	34 (13.2)	
ASA physical status, *n* (%)	0.015			
I–II	293 (68.9)	98 (58.3)	195 (75.9)	
III–IV	132 (31.1)	70 (41.7)	62 (24.1)	
Planned surgical category, *n* (%)	0.09			
General surgery	186 (43.8)	81 (48.2)	105 (40.9)	
Orthopedic surgery	104 (24.5)	33 (19.6)	71 (27.6)	
Gynecologic/urologic	135 (31.8)	54 (32.1)	81 (31.5)	
Expected operative time, min (median, IQR)	165 (120–240)	180 (130–260)	150 (115–220)	0.003
Sleep quality score (mean ± SD)	6.8 ± 3.1	8.1 ± 3.0	5.9 ± 2.8	<0.001
Insomnia symptoms, *n* (%)	147 (34.6)	75 (44.6)	72 (28.0)	<0.001
Depressive symptoms (SDS score) (mean ± SD)	44.3 ± 10.8	50.1 ± 11.0	40.3 ± 8.9	<0.001
Pain at admission (NRS) (median, IQR)	3 (2–5)	4 (2–5)	3 (1–4)	0.002
Night-time smartphone use ≥ 1 h, *n* (%)	196 (46.1)	99 (58.9)	97 (37.7)	<0.001
Daily smartphone use, hours (mean ± SD)	3.8 ± 2.1	4.6 ± 2.3	3.3 ± 1.8	<0.001
Social support score (mean ± SD)	33.8 ± 7.2	31.2 ± 6.9	35.5 ± 7.0	<0.001
Trait anxiety score (baseline) (mean ± SD)	42.1 ± 9.6	48.9 ± 9.2	37.6 ± 7.8	<0.001

BMI = Body mass index; SD = Standard deviation; ASA = American Society of Anesthesiologists physical status classification; IQR = Interquartile range; SDS = Self-Rating Depression Scale; NRS = Numerical rating scale. *p*-values were calculated using independent-samples *t-*test or Mann–Whitney *U* test for continuous variables and χ^2^ test for categorical variables. Preoperative anxiety was defined according to standardized anxiety scale cutoffs.

### Univariate analysis of factors associated with preoperative anxiety

The results of the univariate logistic regression analyses examining factors associated with preoperative anxiety are presented in Table 2. Increasing age was significantly associated with a lower likelihood of preoperative anxiety (odds ratio [OR] per year increase, 0.97; 95% confidence interval [CI], 0.96–0.99; *p* < 0.001). Female sex was associated with more than a twofold higher odds of anxiety compared with male patients (OR, 2.04; 95% CI, 1.38–3.01; p < 0.001). Higher body mass index (BMI) was inversely associated with preoperative anxiety (OR per kg/m^2^, 0.90; 95% CI, 0.84–0.96; *p* = 0.002). Educational level was also significantly associated with anxiety (*p* = 0.015), with patients who had a college education or above showing a lower risk compared with those with primary education or below. Among clinical characteristics, hypertension (OR, 0.55; 95% CI, 0.36–0.86; *p* = 0.008) and cardiovascular disease (OR, 0.54; 95% CI, 0.29–0.98; *p* = 0.045) were associated with lower odds of anxiety, whereas a history of previous surgery was associated with increased odds (OR, 1.50; 95% CI, 1.02–2.20; p = 0.04). Diabetes mellitus, smoking status, and regular alcohol consumption were not significantly associated with preoperative anxiety. Perioperative factors also showed significant associations. Patients with a higher ASA physical status (III–IV) had increased odds of anxiety compared with those classified as ASA I–II (OR, 2.17; 95% CI, 1.45–3.25; *p* < 0.001). Expected operative time was positively associated with anxiety (OR per 30-min increase, 1.18; 95% CI, 1.06–1.31; *p* = 0.002), whereas planned surgical category was not significantly related to anxiety status. Psychological, behavioral, and social variables demonstrated strong associations with preoperative anxiety. Poorer sleep quality and the presence of insomnia symptoms were both significantly associated with higher odds of anxiety (sleep quality score: OR per point, 1.25; 95% CI, 1.17–1.34; insomnia symptoms: OR, 2.08; 95% CI, 1.39–3.10; both *p* < 0.001). Higher depressive symptom scores and greater pain intensity at admission were also associated with increased odds of anxiety. In addition, night-time smartphone use for at least 1 h and longer daily smartphone use were both strongly associated with preoperative anxiety. In contrast, higher social support scores were associated with a reduced likelihood of anxiety (OR per point, 0.92; 95% CI, 0.89–0.95; *p* < 0.001). Baseline trait anxiety showed a strong positive association with preoperative anxiety (OR per point, 1.13; 95% CI, 1.10–1.16; *p* < 0.001).

**Table 2:** Univariate analysis of factors associated with preoperative anxiety among surgical patients (*N* = 425).

Variables	OR	95% CI	*P-*value
Age (per year increase)	0.97	0.96–0.99	<0.001
Female sex	2.04	1.38–3.01	<0.001
BMI (per kg/m^2^)	0.90	0.84–0.96	0.002
Education level	0.015		
Secondary vs. primary	0.77	0.48–1.23	0.27
College vs. primary	0.52	0.31–0.86	0.011
Hypertension	0.55	0.36–0.86	0.008
Diabetes mellitus	0.64	0.36–1.15	0.13
Cardiovascular disease	0.54	0.29–0.98	0.045
Previous surgery	1.50	1.02–2.20	0.04
Current smoker	0.69	0.37–1.26	0.22
Regular alcohol use	0.59	0.31–1.12	0.11
ASA classification (III–IV vs I–II)	2.17	1.45–3.25	<0.001
Planned surgical category	0.12		
Orthopedic vs. general surgery	0.68	0.41–1.13	0.13
Gynecologic/urologic vs. general	0.88	0.56–1.39	0.59
Expected operative time (per 30 min)	1.18	1.06–1.31	0.002
Sleep quality score (per point)	1.25	1.17–1.34	<0.001
Insomnia symptoms	2.08	1.39–3.10	<0.001
Depressive symptoms (SDS score, per point)	1.10	1.07–1.13	<0.001
Pain at admission (NRS, per point)	1.17	1.05–1.31	0.005
Night-time smartphone use ≥1 h	2.41	1.63–3.56	<0.001
Daily smartphone use (per hour)	1.32	1.18–1.48	<0.001
Social support score (per point)	0.92	0.89–0.95	<0.001
Trait anxiety score (per point)	1.13	1.10–1.16	<0.001

OR = Odds ratio; CI = Confidence interval; BMI = Body mass index; ASA = American Society of Anesthesiologists physical status classification; SDS = Self-Rating Depression Scale; NRS = Numerical rating scale. Univariate logistic regression analyses were performed to estimate the association between each predictor and preoperative anxiety. Continuous variables were modeled per unit increase unless otherwise specified. A *P-*value <0.05 was considered statistically significant.

### Multivariable analysis of independent predictors of preoperative anxiety

The results of the multivariable logistic regression analysis identifying independent predictors of preoperative anxiety are presented in Table 3. After adjustment for potential confounding factors, several demographic, clinical, psychological, and behavioral variables remained independently associated with preoperative anxiety. Although history of previous surgery was associated with preoperative anxiety in the univariate analysis, it did not retain independent predictive value after multivariable adjustment and therefore was not included in the final model. Female sex was associated with a significantly higher likelihood of anxiety compared with male patients (adjusted odds ratio [aOR], 1.78; 95% confidence interval [CI], 1.12–2.84; *p* = 0.016). Lower body mass index was independently associated with increased odds of anxiety (aOR per kg/m^2^ decrease, 0.93; 95% CI, 0.86–0.99; *p* = 0.032). From a perioperative perspective, patients with a higher ASA physical status (III–IV) had increased odds of preoperative anxiety compared with those classified as ASA I–II (aOR, 1.89; 95% CI, 1.19–3.02; *p* = 0.007). Longer expected operative time was also independently associated with anxiety (aOR per 30-min increase, 1.12; 95% CI, 1.01–1.25; *p* = 0.031). Psychological and behavioral factors showed robust independent associations with preoperative anxiety. Poorer sleep quality (aOR per point increase, 1.17; 95% CI, 1.08–1.27; *p* < 0.001) and higher depressive symptom scores (aOR per point increase, 1.06; 95% CI, 1.03–1.09; p < 0.001) were both significant risk factors. In addition, night-time smartphone use for at least 1 h (aOR, 1.72; 95% CI, 1.10–2.69; *p* = 0.017) and longer daily smartphone use (aOR per hour increase, 1.18; 95% CI, 1.05–1.34; *p* = 0.006) remained independently associated with higher odds of anxiety. Conversely, greater social support was independently associated with a lower likelihood of preoperative anxiety (aOR per point increase, 0.95; 95% CI, 0.92–0.98; *p* = 0.002). Baseline trait anxiety demonstrated a strong and independent association with preoperative anxiety (aOR per point increase, 1.08; 95% CI, 1.05–1.11; *p* < 0.001).

**Table 3:** Multivariable logistic regression analysis identifying independent predictors of preoperative anxiety among surgical patients (*N* = 425).

Independent predictors	Adjusted OR	95% CI	*P-*value
Female sex	1.78	1.12–2.84	0.016
BMI (per kg/m^2^ decrease)	0.93	0.86–0.99	0.032
ASA classification (III–IV)	1.89	1.19–3.02	0.007
Expected operative time (per 30 min)	1.12	1.01–1.25	0.031
Sleep quality score (per point)	1.17	1.08–1.27	<0.001
Depressive symptoms (SDS score, per point)	1.06	1.03–1.09	<0.001
Night-time smartphone use ≥1 h	1.72	1.10–2.69	0.017
Daily smartphone use (per hour)	1.18	1.05–1.34	0.006
Social support score (per point)	0.95	0.92–0.98	0.002
Trait anxiety score (per point)	1.08	1.05–1.11	<0.001

OR = Odds ratio; CI = Confidence interval; BMI = Body mass index; ASA = American Society of Anesthesiologists physical status classification; SDS = Self-Rating Depression Scale. Variables included in the multivariable logistic regression model were selected based on clinical relevance and statistical significance in univariate analyses. Adjusted odds ratios represent the independent association between each predictor and preoperative anxiety while controlling for all other variables in the model. Continuous variables were modeled per unit increase unless otherwise noted. A two-sided *P-*value <0.05 was considered statistically significant.

### Model performance and validation

**Figure 1:**
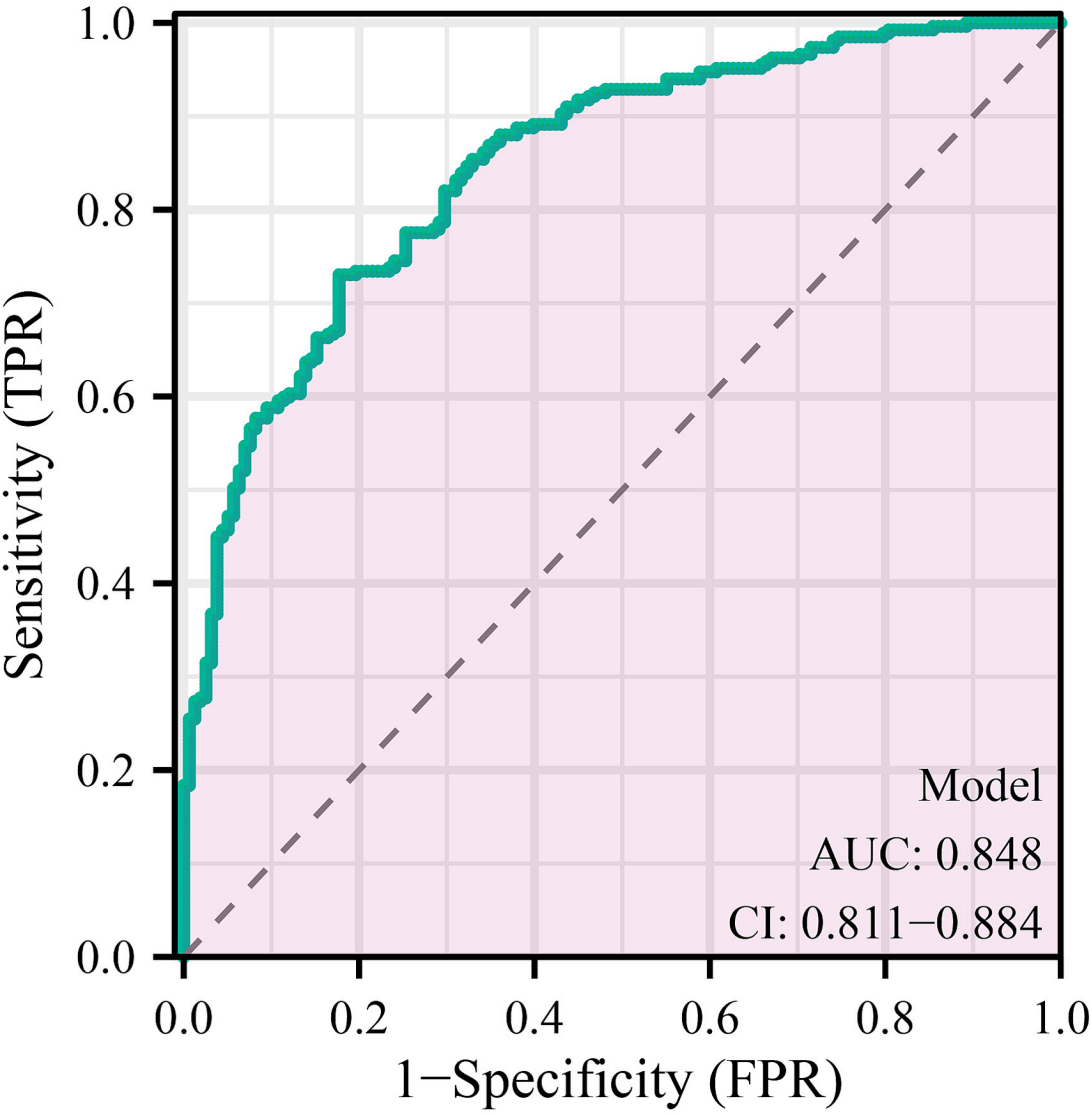
Receiver operating characteristic (ROC) curve of the multivariable prediction model for preoperative anxiety. The receiver operating characteristic (ROC) curve illustrates the discriminative performance of the multivariable prediction model for preoperative anxiety. The area under the curve (AUC) was 0.848, with a 95% confidence interval of 0.811–0.884, indicating good discrimination between patients with and without preoperative anxiety. The diagonal dashed line represents the reference line corresponding to random prediction.

**Figure 2:**
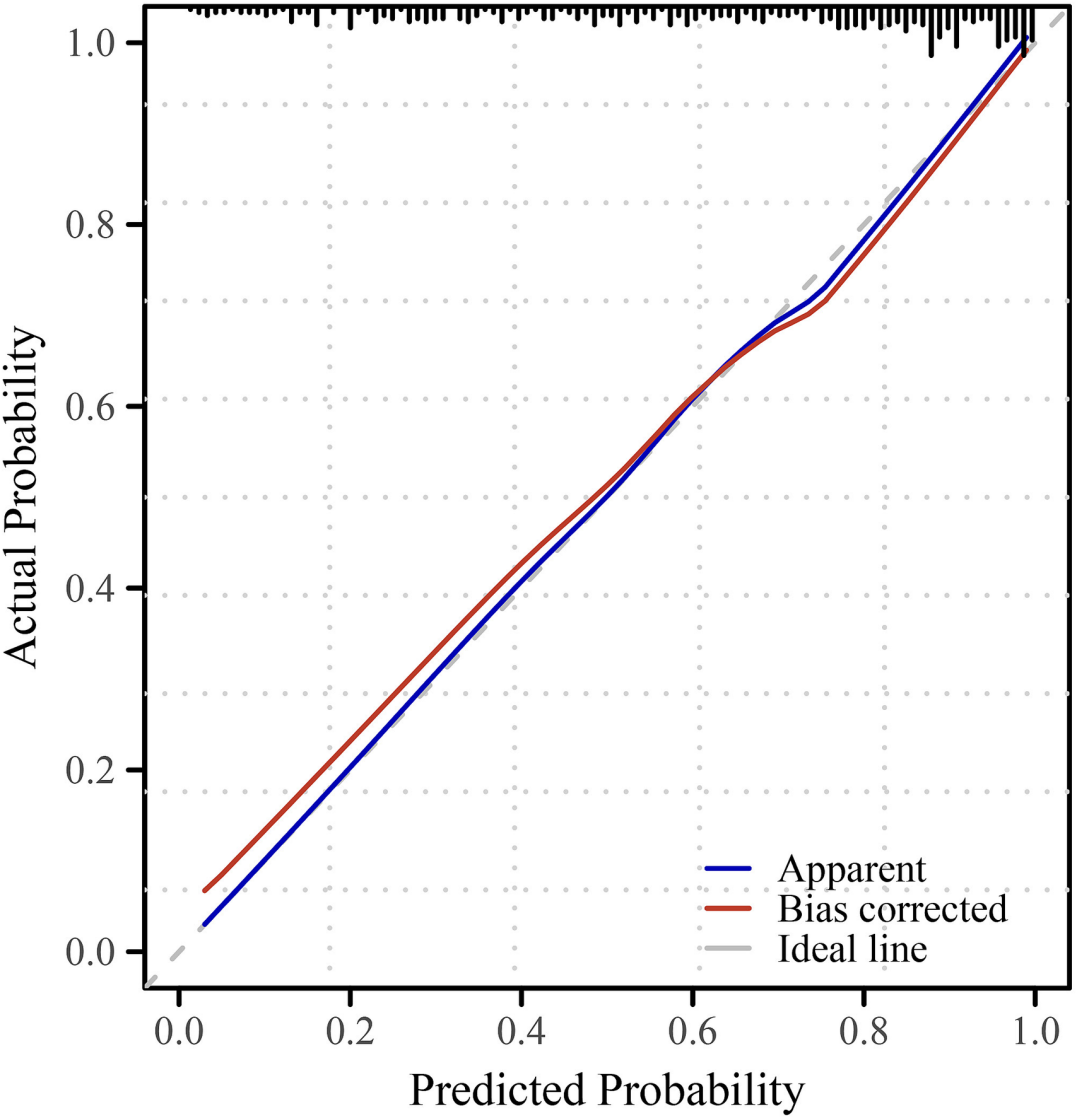
Calibration plot of the multivariable prediction model for preoperative anxiety. The calibration plot shows the agreement between predicted probabilities and observed anxiety status for preoperative anxiety. The apparent curve represents the model’s original performance, while the bias-corrected curve was obtained using internal validation. The dashed diagonal line indicates perfect calibration. The close alignment of the bias-corrected curve with the ideal line suggests good calibration of the prediction model.

**Figure 3:**
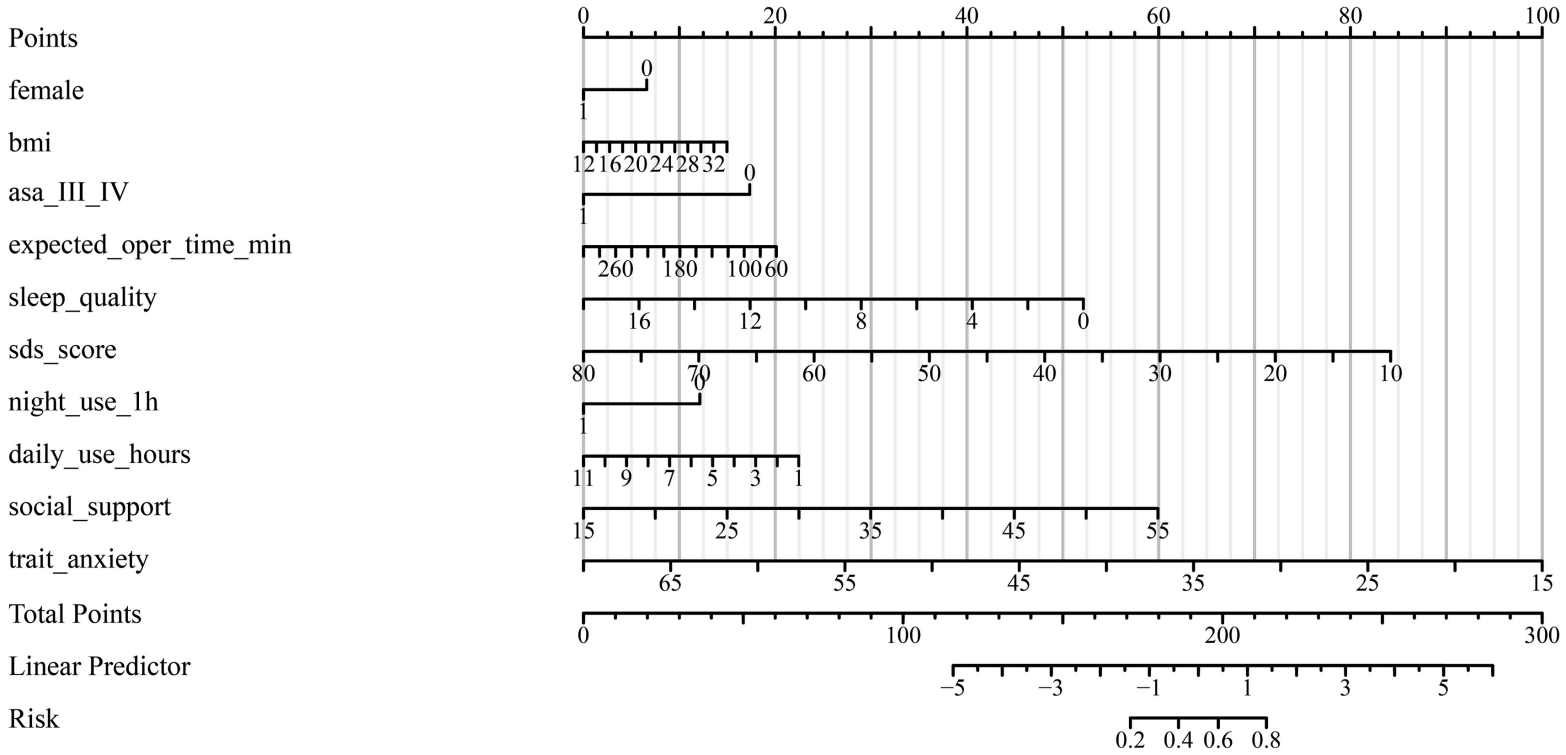
Nomogram for individualized prediction of preoperative anxiety. The nomogram was constructed based on the multivariable logistic regression model to estimate the individualized risk of preoperative anxiety. Each predictor is assigned a corresponding point value according to its relative contribution to the model. The total score is calculated by summing the points for all predictors, which can then be used to determine the predicted probability of preoperative anxiety.

The discriminative performance of the multivariable prediction model for preoperative anxiety was evaluated using receiver operating characteristic (ROC) analysis. The ROC curve demonstrated good discrimination, with an area under the curve (AUC) of 0.848 (95% confidence interval [CI], 0.811–0.884), indicating a strong ability of the model to distinguish between patients with and without preoperative anxiety (Figure 1). To assess the impact of sample heterogeneity, subgroup analyses were conducted according to prespecified clinical strata. The model demonstrated stable discriminative performance across surgical categories, ASA physical status strata, and sex, with subgroup-specific AUC estimates showing moderate variability and overlapping confidence intervals (Supplementary Table S1). Model calibration was assessed using calibration plots. The bias-corrected calibration curve showed good agreement between predicted probabilities and observed anxiety status across the range of risk, closely approximating the ideal reference line, which suggested adequate calibration and limited overfitting of the model (Figure 2). Based on the multivariable logistic regression model, a nomogram was constructed to facilitate individualized risk estimation of preoperative anxiety by integrating demographic, clinical, psychological, and behavioral predictors (Figure 3). By summing the points assigned to each predictor, clinicians can obtain an individualized predicted probability of preoperative anxiety for each patient. At the optimal probability threshold determined using the Youden index, the model achieved a sensitivity of 0.73 and a specificity of 0.82, with an overall accuracy of 0.76 in the overall cohort. Similar discriminative patterns were observed across major subgroups, supporting the robustness of the model in heterogeneous surgical populations. The corresponding Youden index was 0.55, supporting a favorable balance between sensitivity and specificity at this cut-off value of 0.70 (Table 4).

**Table 4:** Discriminative performance of the predictive model for preoperative anxiety.

**Model**	**AUC (95% CI)**	**Optimal cut-off**	**Sensitivity**	**Specificity**	**Accuracy**	**Youden index**
Multivariable model	0.848 (0.811–0.884)	0.70	0.73	0.82	0.76	0.55

The optimal cut-off value was determined using the Youden index. Sensitivity and specificity correspond to the probability threshold that maximized the sum of sensitivity and specificity. AUC indicates the discriminative ability of the multivariable prediction model.

## Discussion

In this retrospective observational study, we identified a set of demographics, clinical, psychological, behavioral, and social factors independently associated with preoperative anxiety and developed a multivariable prediction model to estimate individualized risk. Consistent with the study objective, the model integrated routinely available preoperative data and demonstrated good discriminative ability, with an AUC of 0.848, as well as adequate calibration. A nomogram was further constructed to facilitate practical application of the model in clinical settings.

Female sex, higher ASA physical status, longer expected operative time, poorer sleep quality, higher depressive symptom scores, night-time smartphone use, longer daily smartphone use, and elevated trait anxiety were identified as independent risk factors for preoperative anxiety, whereas greater social support and higher body mass index were independently protective. These findings underscore the multifactorial nature of preoperative anxiety and highlight the importance of simultaneously considering clinical vulnerability, psychological predisposition, behavioral patterns, and social resources when assessing perioperative mental health risk.

In the overall cohort, increasing age was associated with a lower likelihood of preoperative anxiety in the univariate analysis. This finding may reflect several factors, including greater prior exposure to healthcare, different expectations regarding surgery, or age-related differences in emotional appraisal and coping. However, this pattern should not be interpreted as uniformly applicable to all older adults. In elderly patients, additional geriatric vulnerabilities—particularly frailty, multimorbidity, reduced physiological reserve, cognitive vulnerability, and fear of postoperative functional decline or loss of independence—may meaningfully influence preoperative emotional responses. Accordingly, the relationship between age and anxiety may be more heterogeneous in older populations than in the overall adult cohort. Because frailty was not routinely measured using a standardized instrument in this retrospective dataset, we were unable to determine whether the observed age-related pattern differed across robust versus frail older adults. This issue warrants dedicated evaluation in future geriatric-focused studies.

Consistent with previous literature, female sex and higher surgical risk, reflected by ASA physical status III–IV and longer expected operative time, were independently associated with an increased likelihood of preoperative anxiety (14, 16). Female patients have been repeatedly shown to report higher levels of perioperative anxiety, potentially due to differences in emotional processing, stress perception, and coping strategies (6). Higher ASA classification and prolonged operative time may increase perceived uncertainty and fear related to surgical and anesthetic risks, thereby exacerbating anxiety before surgery (17).

A history of previous surgery showed a modest association with preoperative anxiety in the univariate analysis, but this variable was not retained in the final multivariable model. One possible explanation is that prior surgical experience may not exert a uniform effect on preoperative anxiety; depending on the individual context, it may either reduce uncertainty through familiarity or increase distress through negative prior experiences. In the present cohort, its apparent univariate association may have been attenuated after adjustment for stronger coexisting clinical and psychosocial predictors, and therefore it did not demonstrate sufficient independent predictive value for inclusion in the final model or nomogram.

Psychological factors emerged as strong independent predictors in the present model. Poor sleep quality, higher depressive symptom scores, and elevated trait anxiety were all significantly associated with preoperative anxiety. These findings align with existing evidence indicating that sleep disturbance and depressive symptoms contribute to emotional dysregulation and heightened stress reactivity in perioperative settings (18, 19). Trait anxiety, representing a stable predisposition to perceive situations as threatening, has been consistently recognized as a key determinant of situational anxiety, including anxiety related to surgery and anesthesia (20). The integration of these psychological variables substantially enhanced the predictive performance of the model.

Notably, behavioral factors related to smartphone use were independently associated with preoperative anxiety. Patients reporting night-time smartphone use for at least 1 h and longer daily smartphone use duration had a higher risk of anxiety, even after adjustment for sleep quality and depressive symptoms. Previous studies in non-surgical populations have shown that excessive smartphone use, particularly during night-time, is associated with circadian rhythm disruption, impaired sleep, and increased anxiety symptoms (21, 22). Our findings extend this evidence to surgical patients, suggesting that digital behaviors may represent a modifiable risk factor for preoperative anxiety. This highlights the potential value of incorporating simple behavioral assessments into routine preoperative evaluation.

In contrast, greater social support was independently protective against preoperative anxiety. Social support has long been recognized as a buffering factor against stress and emotional distress, particularly in medical and surgical contexts (23). Patients with stronger perceived social support may experience reduced uncertainty and greater reassurance before surgery, thereby mitigating anxiety. This finding underscores the importance of psychosocial resources in perioperative mental health and supports the inclusion of social support measures in risk assessment models.

An additional point that warrants careful consideration is the role of educational attainment and its potential relationship with health literacy. In the univariate analysis, higher education was associated with lower odds of preoperative anxiety, but this association did not remain statistically significant in the final multivariable model. One possible explanation is that education may partly act as a proxy for health literacy, which may influence a patient’s ability to understand perioperative information, appraise procedural risk, and cope with uncertainty before surgery (8). However, health literacy was not directly measured in this retrospective cohort; therefore, this interpretation should be considered hypothesis-generating rather than conclusive. It is also possible that the apparent univariate effect of education was partly explained by other correlated psychosocial variables retained in the final model, such as depressive symptoms, sleep quality, trait anxiety, or perceived social support. Future prospective studies incorporating validated health literacy instruments are needed to clarify whether health literacy exerts an independent effect on preoperative anxiety beyond formal educational attainment.

Compared with prior studies that primarily focused on individual risk factors or relied on univariate analyses (13, 24, 25), the present study offers a comprehensive multivariable approach that integrates clinical, psychological, behavioral, and social domains. The use of a nomogram allows for intuitive visualization of individual risk contributions and may facilitate clinical implementation. By enabling early identification of high-risk patients, this model may support targeted psychological screening, preoperative counseling, and behavioral interventions aimed at reducing anxiety and improving perioperative outcomes.

Several limitations of this study should be acknowledged. First, the retrospective design may introduce selection bias and limits causal inference. In addition, the analytic cohort was restricted to patients with routinely documented preoperative psychological assessment data; therefore, patients without such documentation were not captured, which may limit representativeness and introduce selection bias. Although consecutive eligible patients were retrospectively identified and included to minimize selection bias, unmeasured confounding cannot be fully excluded. Second, psychological and behavioral variables, including sleep quality, depressive symptoms, smartphone use, and social support, were derived from standardized self-reported assessments documented in routine clinical practice; although these measures were collected using a uniform preoperative assessment process, they may still be subject to reporting bias or misclassification. In addition, health literacy was not directly assessed using a validated instrument in the present cohort. As a result, we could not determine whether the observed association of educational level in univariate analysis partly reflected underlying differences in health literacy, which represents a source of residual unmeasured confounding. Future prospective studies should incorporate standardized health literacy measures to better disentangle the respective roles of educational attainment and health literacy in shaping preoperative anxiety risk. Another methodological consideration is that dichotomization of preoperative anxiety may have resulted in some loss of information compared with modeling anxiety as a continuous outcome; however, this approach was chosen to enhance clinical interpretability and feasibility for screening purposes. Such bias could potentially lead to underestimation or overestimation of the strength of associations. Third, the study was conducted at a single tertiary teaching hospital, and institutional practices or patient characteristics may differ from those in other settings. Moreover, although we adjusted for commonly used perioperative proxies of clinical severity and procedural complexity (e.g., ASA physical status and expected operative time), more granular diagnosis-specific severity measures (such as disease staging, frailty indices, objective laboratory markers, or validated surgical risk scores) were not uniformly available in this retrospective dataset; therefore, residual confounding related to underlying illness severity cannot be fully excluded. This limitation is particularly relevant to interpretation of the inverse age–anxiety association observed in the overall cohort, because frailty and other geriatric vulnerabilities may modify anxiety risk among older adults in ways not captured by chronological age alone. In particular, as a tertiary center, our institution may manage a relatively selected case mix with higher perioperative complexity, which may further limit representativeness compared with lower-acuity surgical populations. In addition, postoperative surgical outcomes (e.g., complications, recovery metrics, or length of stay) were not modeled in this study. Future prospective and multicenter studies are needed to evaluate whether early identification of high-risk patients and targeted preoperative psychological support based on this risk stratification approach translate into improved perioperative outcomes. Fourth, although the number of patients meeting the criteria for preoperative anxiety yielded an adequate number of target-condition events to support multivariable modeling, the prediction model was developed and evaluated within the same single-center cohort and underwent internal validation only; external validation in independent hospitals or geographically distinct cohorts was not performed. Therefore, whether the model would retain comparable discrimination, calibration, and clinical reliability across different institutions, perioperative workflows, or patient populations remains unconfirmed. This limitation restricts the generalizability and transportability of the model and underscores the need for external validation before broader clinical implementation. Finally, multiple candidate predictors were evaluated, and although variables were selected based on clinical relevance and prior literature, the possibility of overfitting cannot be entirely excluded.

Despite these limitations, the findings of this study are consistent with and extend existing evidence on preoperative anxiety. Previous studies have reported associations between preoperative anxiety and female sex, surgical risk, sleep disturbance, depressive symptoms, and trait anxiety (26, 27). Our results corroborate these associations and further demonstrate that integrating these factors into a multivariable model substantially improves risk discrimination compared with consideration of individual predictors alone. Importantly, this study adds to the literature by highlighting the independent contribution of smartphone use behaviors to preoperative anxiety. While excessive smartphone use has been linked to anxiety and sleep disruption in general populations (28, 29), evidence in surgical patients has been scarce. The persistence of this association after adjustment for sleep quality and depressive symptoms suggests that smartphone use may reflect broader patterns of behavioral dysregulation or coping strategies under stress. In contrast, the protective role of social support observed in this study aligns with stress-buffering theories and prior clinical evidence emphasizing the importance of psychosocial resources in surgical care (30, 31).

Taken together, these findings support a cautious interpretation that preoperative anxiety arises from the interaction of stable psychological traits, modifiable behavioral factors, and perioperative clinical stressors. Although anxiety is inherently a continuous construct, the use of a dichotomized anxiety status in this study reflects a pragmatic, clinically oriented approach to risk stratification rather than an attempt to model symptom severity across the full spectrum. Importantly, in the present study, preoperative anxiety was not conceptualized as a postoperative surgical outcome, but rather as a target condition for preoperative screening and risk stratification, and no causal inferences regarding surgical outcomes should be drawn from the model, particularly given the possibility of residual confounding related to underlying illness severity. The developed model should therefore be viewed as a tool for risk stratification rather than a diagnostic instrument, and its use should complement, rather than replace, clinical judgment. Accordingly, the methodological framework and analytical strategy of this study are intended to support hypothesis-informed risk stratification within clearly defined clinical boundaries, rather than to provide definitive causal or outcome-based conclusions.

The generalizability of the present findings should be interpreted with appropriate caution. Because the model was both developed and internally validated within a single tertiary teaching hospital, its performance in other hospital settings, healthcare systems, or patient populations remains uncertain. Differences in case mix, referral patterns, perioperative workflows, and institutional practice structures may affect transportability and limit the clinical reliability of the model outside the derivation setting. The study population comprised adult patients undergoing elective surgery at a single tertiary hospital in Southwest China, and institutional characteristics, case mix, and perioperative practices may differ from those of other healthcare systems, emergency surgical settings, or populations with distinct sociodemographic profiles. Importantly, the heterogeneity inherent to the study cohort reflects routine perioperative practice rather than a restricted or highly selected population. To explore internal robustness, we performed prespecified subgroup analyses across major surgical categories, ASA strata, and sex, which suggested broadly consistent discrimination within our cohort; however, these analyses do not constitute external validation and should not be interpreted as evidence of transportability across institutions. Moreover, most predictors incorporated into the model—including ASA physical status, expected operative time, sleep quality, depressive symptoms, smartphone use behaviors, and social support—are routinely assessed or readily obtainable during standard preoperative evaluation. While external validation in independent cohorts is required prior to widespread clinical implementation, the conceptual framework and modeling strategy may be informative for broader surgical populations, but their practical applicability should not be assumed without formal external testing. Future multicenter studies—including prospective cohorts and geographically diverse hospitals—are warranted to externally validate the model, quantify calibration drift, and assess whether local recalibration is required before implementation beyond similar settings.

In this retrospective observational study, we developed and internally validated a multivariable prediction model for preoperative anxiety using routinely available preoperative data. The model demonstrated good discriminative ability and calibration, and the derived nomogram provides a practical tool for individualized risk assessment in surgical patients. By integrating demographic, clinical, psychological, behavioral, and social factors, including sleep quality, depressive symptoms, smartphone use behaviors, and social support, this model may facilitate early identification of patients at high risk of preoperative anxiety. Future multicenter studies with external validation are warranted to confirm the generalizability of the model and to evaluate its potential role in guiding targeted perioperative psychological interventions.

## Data Availability

The original contributions presented in the study are included in the article/Supplementary material, further inquiries can be directed to the corresponding author/s.
