# Supplementary material for: Development and internal validation of a multivariable risk stratification model for preoperative anxiety in surgical patients: a retrospective observational study
Source: Front Med (Lausanne). 2026 Apr 21;13:1798841. doi: 10.3389/fmed.2026.1798841 (PMC13139159; doi:10.3389/fmed.2026.1798841)
Supplement: Supplementary file 1 [file Table_1.docx]

**Table S1.** Subgroup-specific discriminative performance of the multivariable prediction model for preoperative anxiety across prespecified clinical strata.

| **Predictor** | **VIF** | **Tolerance** |
| --- | --- | --- |
| Female sex | 1.18 | 0.85 |
| BMI | 1.24 | 0.81 |
| ASA physical status (III–IV vs I–II) | 1.33 | 0.75 |
| Expected operative time | 1.41 | 0.71 |
| Sleep quality score | 2.12 | 0.47 |
| Depressive symptoms (SDS score) | 2.31 | 0.43 |
| Night-time smartphone use ≥1 hour | 1.67 | 0.60 |
| Daily smartphone use (hours) | 1.84 | 0.54 |
| Social support score | 1.76 | 0.57 |
| Trait anxiety score | 2.64 | 0.38 |

**Note.** VIF = variance inflation factor. Tolerance was calculated as 1/VIF. No predictor showed evidence of problematic multicollinearity, as all VIF values were below the prespecified threshold of 5.0.
