# Supplementary material for: Development and internal validation of a multivariable risk stratification model for preoperative anxiety in surgical patients: a retrospective observational study
Source: Front Med (Lausanne). 2026 Apr 21;13:1798841. doi: 10.3389/fmed.2026.1798841 (PMC13139159; doi:10.3389/fmed.2026.1798841)
Supplement: Supplementary file 2 [file Table_2.docx]

**Table S2.** Discriminative performance of the multivariable prediction model across prespecified clinical subgroups

| **Subgroup** | **N** | **Anxiety cases, n (%)** | **AUC (95% CI)** |
| --- | --- | --- | --- |
| Overall cohort | 425 | 168 (39.5) | 0.848 (0.811–0.884) |
| **Surgical category** |  |  |  |
| General surgery | 186 | 81 (43.5) | 0.83 (0.78–0.88) |
| Orthopedic surgery | 104 | 33 (31.7) | 0.80 (0.72–0.88) |
| Gynecologic/urologic | 135 | 54 (40.0) | 0.86 (0.81–0.91) |
| **ASA physical status** |  |  |  |
| ASA I–II | 293 | 98 (33.4) | 0.82 (0.77–0.87) |
| ASA III–IV | 132 | 70 (53.0) | 0.87 (0.82–0.92) |
| **Sex** |  |  |  |
| Male | 187 | 56 (29.9) | 0.81 (0.75–0.88) |
| Female | 238 | 112 (47.1) | 0.85 (0.80–0.90) |

**Note.** AUC = area under the receiver operating characteristic curve; CI = confidence interval; ASA = American Society of Anesthesiologists physical status. Subgroup analyses were prespecified to evaluate the robustness of the prediction model across heterogeneous surgical populations. AUCs with 95% confidence intervals were estimated within each subgroup and compared descriptively with the overall cohort. Overlapping confidence intervals suggest broadly comparable discriminative performance across subgroups.
